# Testes Mass, but Not Sperm Length, Increases with Higher Levels of Polyandry in an Ancient Sex Model

**DOI:** 10.1371/journal.pone.0094135

**Published:** 2014-04-15

**Authors:** David E. Vrech, Paola A. Olivero, Camilo I. Mattoni, Alfredo V. Peretti

**Affiliations:** Institute of Animal Diversity and Ecology (IDEA), Laboratory of Reproductive Biology and Evolution, CONICET - The National University of Cordoba, Cordoba, Argentina; University of Nevada School of Medicine, United States of America

## Abstract

There is strong evidence that polyandrous taxa have evolved relatively larger testes than monogamous relatives. Sperm size may either increase or decrease across species with the risk or intensity of sperm competition. Scorpions represent an ancient direct mode with spermatophore-mediated sperm transfer and are particularly well suited for studies in sperm competition. This work aims to analyze for the first time the variables affecting testes mass, ejaculate volume and sperm length, according with their levels of polyandry, in species belonging to the Neotropical family Bothriuridae. Variables influencing testes mass and sperm length were obtained by model selection analysis using corrected Akaike Information Criterion. Testes mass varied greatly among the seven species analyzed, ranging from 1.6±1.1 mg in *Timogenes dorbignyi* to 16.3±4.5 mg in *Brachistosternus pentheri* with an average of 8.4±5.0 mg in all the species. The relationship between testes mass and body mass was not significant. Body allocation in testes mass, taken as Gonadosomatic Index, was high in *Bothriurus cordubensis* and *Brachistosternus ferrugineus* and low in *Timogenes* species. The best-fitting model for testes mass considered only polyandry as predictor with a positive influence. Model selection showed that body mass influenced sperm length negatively but after correcting for body mass, none of the variables analyzed explained sperm length. Both body mass and testes mass influenced spermatophore volume positively. There was a strong phylogenetic effect on the model containing testes mass. As predicted by the sperm competition theory and according to what happens in other arthropods, testes mass increased in species with higher levels of sperm competition, and influenced positively spermatophore volume, but data was not conclusive for sperm length.

## Introduction

Sperm competition is a widespread phenomenon that influences several sexual characters [1]–[4]. It is defined as the competition of ejaculates of two or more males to fertilize a given set of ova [5]–[7]. In a polyandrous mating system, and when the competition is numeric [6], [7], there is a selective pressure on males to increase investment in sperm production, thus showing relatively larger testes compared to monogamous relatives [7]–[11]. Larger testes may produce more sperm and give the male an advantage in numerical sperm competition [12]. Nevertheless, sperm is costly [13], [14] and males should optimize their investment in ejaculates allocating sperm strategically according to sperm competition risk [6], [7], [15], [16]. Testes size is considered a reliable index of sperm competition [17], but there are other tactics that can affect testes size as well (e.g. [18]–[20]).

Sperm size is another trait that may vary according to sperm competition risk. Sperm changes considerably in size (mainly length) among species and sperm size may either increase ([21] (butterflies); [22] (moths); [23], [24] (fishes); [25] (frogs); [26] (snakes); [27]–[29] (birds); [30], [31] (eutherian mammals); [32] (marsupial mammals)), or decrease (e.g. [33] (fishes)) across species with the risk or intensity of sperm competition.

Among arthropods, sperm competition has been widely studied in insects [5], [7], but spiders have also been a good model for sperm competition [34]–[36]. Classic reviews provided by Austad [37] and Thomas and Zeh [38] focused on the influence of sperm competition in shaping mating strategies in spiders and other arachnids. Unfortunately, studies of the influence of sperm competition over testes mass are still lacking in arachnids.

Scorpions are particularly well suited models for studies on sperm competition. Traditionally, they have been considered to be among the most basal arachnids [39], representing an ancient sex model. Sperm is transferred indirectly to the female genital opening, by means of a sclerotized spermatophore deposited in the substrate by the male [40]. Scorpion spermatozoa are long and commonly transferred as sperm packages [41], [42]. All scorpion species are viviparous [40] and in general females are polyandrous, capable of storing sperm in paired seminal receptacles [43]. Males of some species deposit a genital plug that occludes the female’s genital opening after sperm transfer [44]–[46]. Although some courtship characteristics and spermatozoa have been studied in this order (e.g. [41], [42], [47]–[50]), details of sperm competition mechanisms remain unexplored.

In this context, the present study aims to analyze variables affecting testes mass and sperm length in scorpion species of the Neotropical family Bothriuridae. This is a novel approach in these arachnids. In accordance with sperm competition theory, and assuming that greater testes produce more spermatozoa, we predict a positive association between the risk of sperm competition (measured as level of polyandry) and testes mass due to direct selection arising from sperm competition. The ejaculate, in the form of the spermatophore volume, should increase accordingly. Besides, and according to analyses in other groups, we initially expect sperm length to increase with sperm competition risk to confer individual spermatozoa a competitive advantage in sperm competition.

## Materials and Methods

### Analyzed Species

Adult males of eight scorpion species belonging to the Bothriuridae family were analyzed, as listed below together with capture sites, capture technique and number of males: *Bothriurus bonariensis* (C. L. Koch, 1842), Mendiolaza, Córdoba, Argentina, Ultraviolet light (UV light) (N = 11); *Bothriurus cordubensis* Acosta, 1995, Villa Berna, Córdoba, Argentina, turning rocks during the day (N = 6); *Bothriurus rochensis* San Martín, 1965, Piedras de Afilar, Montevideo, Uruguay, UV light (N = 10); *Brachistosternus ferrugineus* (Thorell, 1876), Chancaní Reserve, Córdoba, Argentina, UV light (N = 18); *Brachistosternus pentheri* Mello-Leitão, 1931, Chancaní Reserve, Córdoba, Argentina, UV light (N = 6); *Timogenes elegans* (Mello-Leitão, 1931) Chancaní Reserve, Córdoba, Argentina, UV light (N = 19); *Timogenes dorbignyi* (Guérin Méneville, 1843) Chancaní Reserve, Córdoba, Argentina, UV light (N = 9) *Urophonius brachycentrus* (Thorell, 1876), Tanti, Córdoba, Argentina, turning rocks during the day (N = 9). In all species, captures were between 2009 and 2012. Detailed data of *voucher specimes* and collection deposition are included as Table S1. The authors confirm that no specific permissions were required for capturing those sample sizes (N<20 individuals per species) in their respective localities and that the study did not involve endangered or protected species. In the laboratory, individuals were kept inside individual plastic boxes (10×13×8 cm) with moistened cotton as water supply and fed with larvae of *Tenebrio molitor* Linné (Insecta, Coleoptera). Data on testes mass was only obtained from recently dissected males. Specimens were euthanized using ethyl ether, and dissections were performed in ethanol 80%.

### Comparative Design

#### Body mass and testes mass (bm, tm)

**Figure 1:**
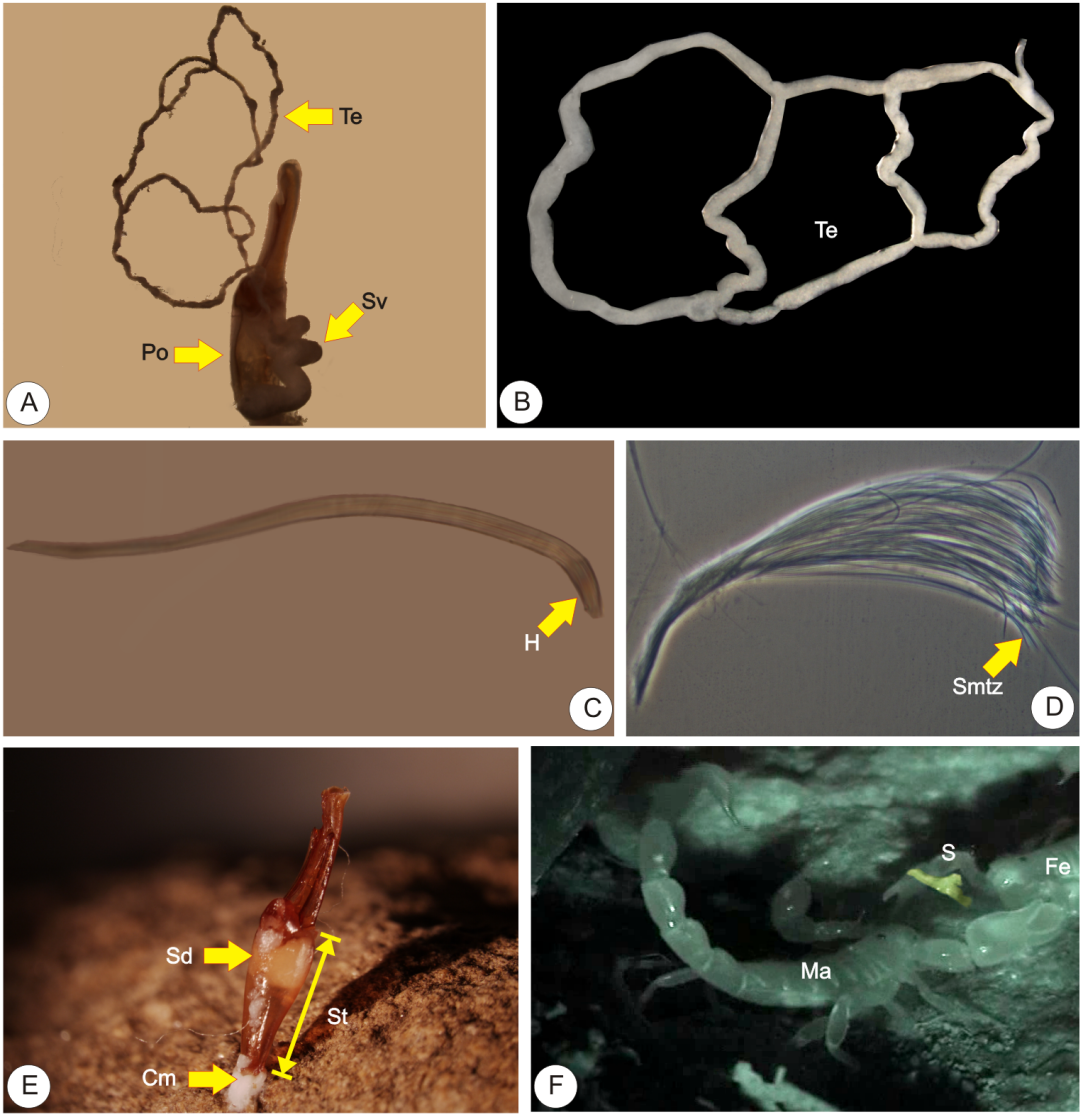
Main reproductive traits in scorpions. A: Right paraxial organ. B: Right testis. C: Sperm package. D: Spermatozoa forming the sperm package. E: Spermatophore deposited in the substrate. F: Mating pair immediately after sperm transfer (the used spermatophore appears in yellow). Abbreviations: Cm: Cementing material which sticks the spermatophore in the substrate; Fe: female; H: Sperm package’s head; where all the spermatozoa heads concur; Ma: male; Po: Paraxial organ; S: Spermatophore; Sd: sperm drop; which is transferred to the female; Smtz: spermatozoa; St: Spermatophore’s trunk; Sv: seminal vesicle; Te: testes.

Specimens were cleaned and dried with tissue paper in order to remove ethanol excess. When dried, they were weighted in a microbalance (Ohaus Pioneer PA114) to the nearest 0.0001 g to obtain the male’s body mass. Males were then dissected under stereoscopic dissection microscope (Nikon SMZ1500). Paired paraxial organs, that produce the hemispermatophores, were removed and testes were cleaned and cut out from them (Figs. 1 A, B). Testes from both paraxial organs were dried from liquid excess using a tissue paper and were weighted together to the nearest 0.0001 g to get the male’s testes mass. In all species, measurements were taken 30 seconds after the value of the microbalance was stabilized. For comparison with other works, we also computed relative testes mass, in the form of a Gonadosomatic Index (GSI: gonad weight/body weight×100) [51]. The growth of testes mass and body mass was described by the ‘allometric relation’ [52], also termed ‘allometric equation’ [53] y =  a*x^b^. This allometric relationship will also be described in the following two variables, sperm length and spermatophore’s volume.

#### Sperm length (sl)

Paraxial organs also contain seminal vesicles [54] (Fig. 1A). Spermatozoa are stored inside seminal vesicles as sperm packages [42], [49] (Fig. 1 C, D). Sperm packages were photographed in a phase contrast microscope (Nikon Eclipse 50i) with an attached digital camera (Nikon DS-fi1). Sperm packages were then measured from the digital images with ImageJ 64 bit software [55]. Sperm packages are elongated and spermatozoa are easily identified (Fig. 1 C, D). Sperm length was measured indirectly as the length of sperm packages per species. Sperm package’s length is considered a reliable measure of sperm length and both measures, spermatozoa and sperm length, correlate [42], [48]. Mean value of sperm package’s length was recorded per male (N = 10 sperm packages per individual) and then averaged per species.

#### Spermatophore’s volume (sv)

Besides testes mass, sperm volume from pre-insemination spermatophores was observed. For this purpose, area and total length of spermatophore’s trunk were taken from one to three males depending on material availability. Volume was estimated using the trunk´s area and its width. We were able to calculate a volume in mm^3^ multiplying the area in mm^2^ by the trunk’s width in mm. The total volume of the spermatophore’s trunk can be taken as an estimative of the sperm volume that is transferred to the female during mating (Fig. 1 E).

#### Risk of sperm competition: polyandry levels (pol)

Polyandry levels were considered as the average number of males a female accepted during the reproductive season, based in controlled laboratory assays [44], [47], [48], [56]–[58] (Fig. 1 F). *Urophonius brachycentrus* is a species whose males deposit a very effective mating plug that occludes the female genital opening [44], [48]. The females only mate once [48], (Costa-Schmidt, Romero-Lebrón, unpublished data). Thus, this species is the only species considered monandrous in this study. In all other species females accepted at least two matings with different males [44], [47], [48], [56]–[58], (Peretti, Vrech, unpublished data) (average number of males accepted per reproductive season: *Timogenes elegans*, 1,5; *T. dorbignyi*, 2; *Bothriurus rochensis*, 2,5; *B. cordubensis*, 2,5; *Brachistosternus ferrugineus*, 2,5; *Br. pentheri*, 3*; B. bonariensis*, 4).

### Statistical Analysis

Measurements were Log_10_ transformed to normalize their distributions (Shapiro-Wilks normality test on Log_10_ transformed data: lbm w = 0.968, p = 0.880; ltm w = 0.889, p = 0.228; lsv w = 0.928, p = 0.502; lsl w = 089, p = 0.263).

We used model selection analysis [59] for choosing among biological meaningful models. All these models were chosen because they could be explained by a hypothesis or an observation in nature. We tested 18 models divided into three categories aiming to explain one response variable in each category. First we tested the effect on testes mass (ltm = Log_10_ Testes mass). The first model was the null model (ltm∼1). This model will have the greatest chance of being chosen when the others models perform poorly in explaining the response variable. This is why the null model was tested with the three response variables. The second model for testes mass was ltm∼lbm for testing the allometric effect of body mass on testes mass. Third, we tested the effect of sperm competition on testes mass by using polyandry levels as a predictor (ltm∼pol). Finally, we used a model assuming an additive effect between these last two variables (ltm∼lbm+pol). If body mass was affecting the response variable, the effect of body mass on this particular variable was controlled for by including body mass in a multiple regression [60]. This multiple regression analysis was performed using a sequential (Type I) sum of squares, in which the predictor variables were added to the model only in the following order: body mass, polyandry to control for the possible effects of body mass. The second set of models was aimed to explain the performance of sperm length. First we tested the null model. Second, as for testes mass, we tested single models with body mass and polyandry as single variables. Here, we added the effect of testes mass on sperm length, as bigger testes could explain the production of larger spermatozoa in some organisms (e.g. [61]). Finally, we added two additive models to test polyandry and testes mass influence with the control of the possible effects of body mass (lsl∼lbm+pol; lsl∼lbm+ltm). The last set of variables tried to explain spermatophore volume. Besides the null model we tested four single models (lsv∼lbm; lsv∼pol; lsv∼ltm, lsv∼lsl). The influence of testes mass is important as a positive influence could be supported by the sperm competition model [17]. The last model, tried to corroborate if bigger spermatozoa influences ejaculate volume. Finally, we tested the influence of the last three variables controlled for body mass as in the other set of models.

We compared these models for each response variable, and we used Akaike’s Information Criterion corrected for small sample size (AICc) to infer the maximum likelihood of the current models, as suggested by Burnham & Anderson [59]. The model with the smallest value of AICc and models whose change in AICc was smaller than 2 (ΔAICc<2) were selected, because this difference suggests substantial evidence for the model (see [63]). It is noteworthy that models with four parameters (y∼a*b) were excluded as their changes in AICc were bigger than 10 (ΔAICc>10). We used Akaike weights to assess the relative strength of the model compared to the other models tested [59]. This value can give the idea of how many times one model is better than the other.

**Figure 2:**
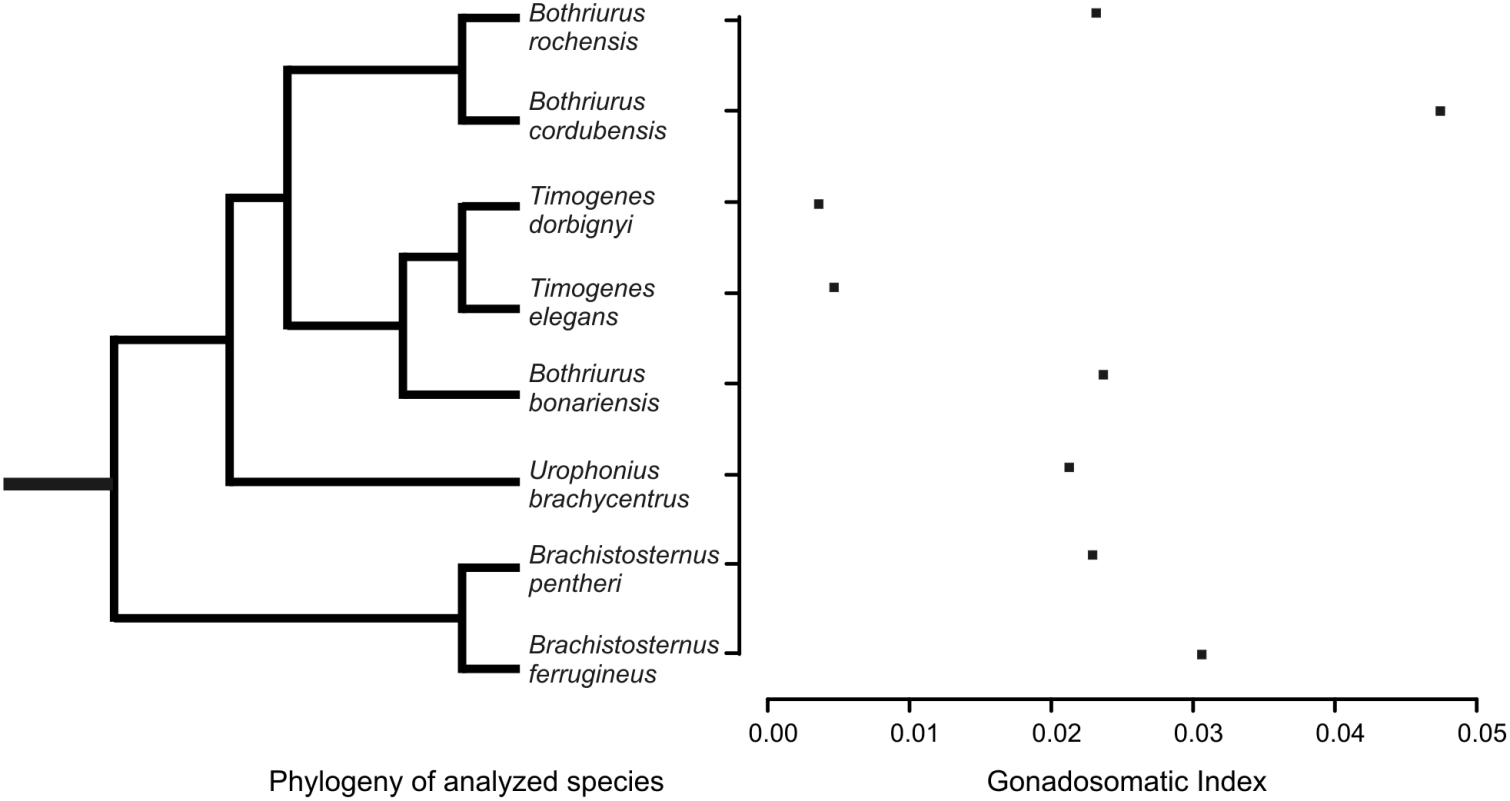
Phylogeny and GSI. Phylogeny of species used in the analysis and their respective Gonadosomatic index values.

Species data may not be free of phylogenetic association. They may share character values because of a common ancestry rather than independent evolution [52], [62]. Because of this lack of independence, regressions were performed using a generalized least-squares approach within a phylogenetic framework (pGLS) [63]. This method estimates a phylogenetic scaling parameter lambda (*λ*), which represents the transformation that makes the data fit a Brownian motion evolutionary model. If *λ* values are close to 0, the variables are likely to have evolved independently of phylogeny, whereas *λ* values close to 1 indicate strong phylogenetic association of the variables. As an advantage, GLS allows a variable degree of phylogenetic control according to each tested model, accounting for differences in the level of phylogenetic association between different traits. The estimation of *λ* values and GLS analyses were performed using a code written by R. Freckleton for the statistical package R v.2.15.1 (R Foundation for Statistical Computing 2012) and the maximum likelihood value of *λ* was compared against models with *λ*  = 1 and *λ*  = 0. Using the current phylogenetic hypotheses available [64], (Mattoni, unpublished data), a cladogram was built for the Bothriuridae studied species (Fig. 2). Branch lengths were assumed equal, thereby assuming a punctuated model of evolution. Data was analyzed with R v. 2.15.1 64 bit open source statistical package [65].

## Results

### Testes Mass, Ejaculate Volume and Sperm Length: Description and Allometric Values

#### Testes mass

Male reproductive allocation differed markedly among the analyzed scorpion species (Table 1). There was an order of magnitude of difference between the species with the lowest testes mass and the species with the highest testes mass values. Testes mass ranged from 1.6±1.1 mg in *Timogenes dorbignyi* to 16.3±4.5 mg in *Brachistosternus pentheri*. The average absolute testes mass among analyzed species was 8.4±5.0 mg. Two species showed low absolute testes mass values (both belonging to *Timogenes*) and two showed high values (*Br. pentheri* and *B. bonariensis*), the rest had intermediate values. *Urophonius brachycentrus*, the species with mating plug, had 3.2±1.4 mg, twice the mass found in *T. dorbignyi*. *Timogenes elegans*, the biggest species in the dataset, had really small testes mass compared to species with big body sizes like *Bothriurus bonariensis* or *Br. pentheri*. In proportion to male’s body size (Gonadosomatic Index, GSI), *B. cordubensis* and *Br. ferrugineus* allocated more in testes mass compared to other analyzed species, in opposition to what happened in both *Timogenes* species which allocated very little in testes mass (see Table 1, Fig. 1).

**Table 1:** Body mass, testes mass, spermatophore volume and sperm length for the scorpion species analyzed in this study.

Species	Body mass (g)	Testes mass (mg)	Spermatophore volume (mm^3^)	Sperm length (µm)	Polyandry	GSI
*Timogenes dorbignyi*	0.43±0.17	1.60±1.10	1.27	229.35±12.49	2.0	0.37%
*Timogenes elegans*	1.55±0.40	7.40±3.70	4.41	240.74±16.37	1.5	0.48%
*Urophonius brachycentrus*	0.15±0.03	3.20±1.40	1.86	301.30±9.10	1.0	2.14%
*Brachistosternus pentheri*	0.71±0.17	16.30±4.50	6.23	207.73±15.02	3.0	2.30%
*Bothriurus rochensis*	0.33±0.03	7.60±4.80	3.37	269.39±15.24	2.5	2.33%
*Bothriurus bonariensis*	0.61±0.13	14.60±7.00	5.66	233.07±12.68	4.0	2.38%
*Brachistosternus ferrugineus*	0.27±0.07	8.40±3.60	3.49	233.04±8.02	2.5	3.07%
*Bothriurus cordubensis*	0.17±0.01	8.20±1.80	3.23	302.39±3.44	2.5	4.75%

These values are given as mean ± standard deviation. Species are ordered by GSI. Polyandry values are the mean number of males a female accepts per mating season in laboratory trials (see text). Abbreviations: GSI: Gonadosomatic Index.

**Figure 3:**
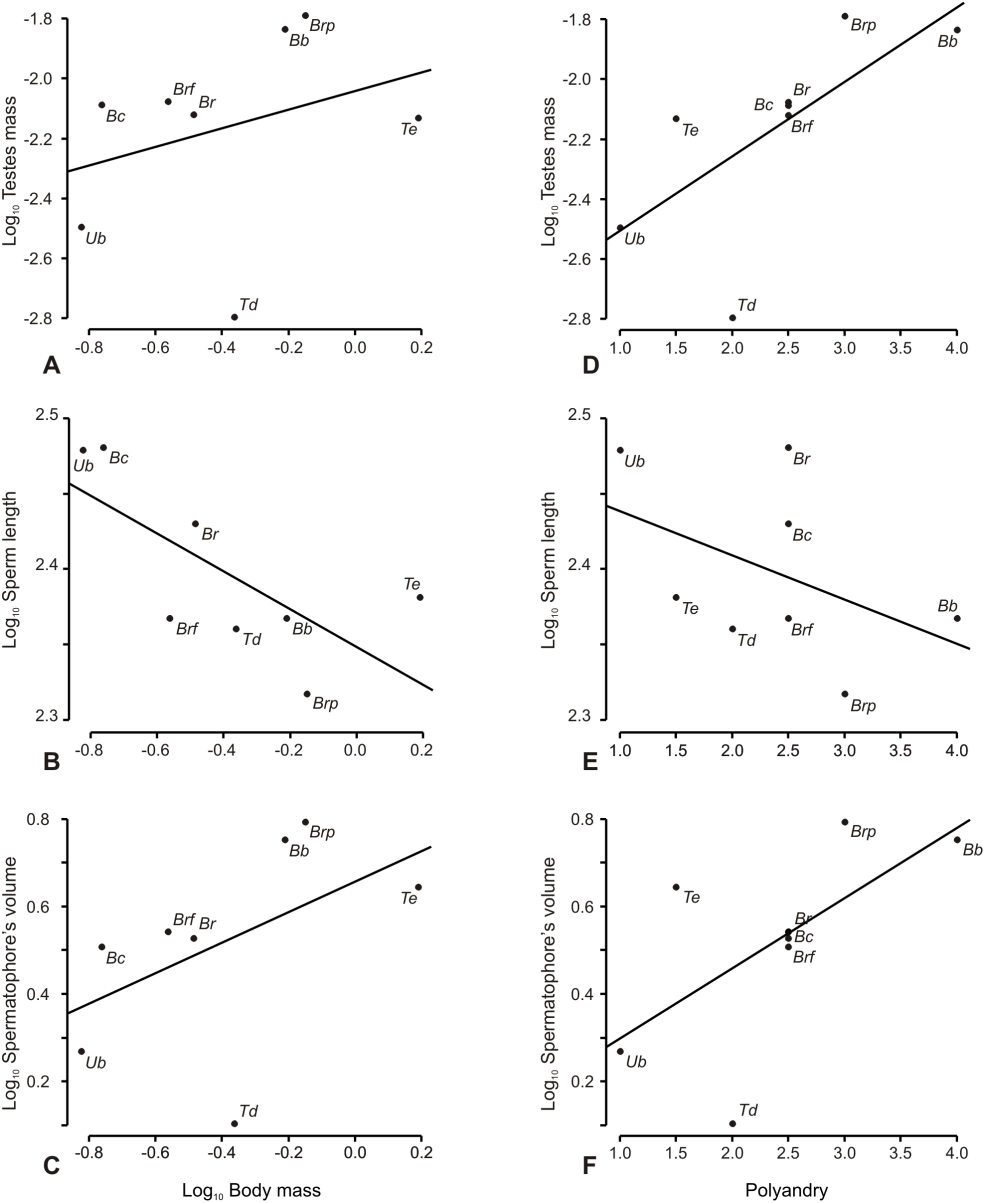
Relationships between variables of sperm competition in bothriurid scorpions. A: Effect of body mass on testes mass. B: Effect of body mass on sperm length. C: Effect of body mass on spermatophore’s volume. D: Effect of polyandry on testes mass. E: Effect of polyandry on sperm length. F: Effect of polyandry on spermatophore’s volume. Abbreviations: Td: *Timogenes dorbignyi*, Te: *Timogenes elegans*, Br: *Bothriurus rochensis*, Bb: *Bothriurus bonariensis*, Bc*: Bothriurus cordubensis*, Brf: *Brachistosternus ferrugineus*, Brp: *Brachistosternus pentheri*, Ub: *Urophonius brachycentrus.*

The linear logarithmic equation tested was Log_10_ (Testes mass)  =  −2.0901+0.2521*Log_10_ (Body mass) (Fig. 3 A). This relationship between testes mass and body mass lacked statistical significance (ltm∼lbm, Table 2).

**Table 2:** Model selection for predictive variables affecting three dependent variables in the eight analyzed species.

Dependent variable	Predictor	Slope	F	p	AICc	ΔAICc	wt	λ
Log_10_ Testes mass	1	−2.14		<0.001	6.75	2.00	0.22	0.0001^††^
	Log_10_ body mass	0.49	2.02	0.21	9.45	5.00	0.06	0.0235^††^
	**Polyandry**	**0.27**	**9.03**	**0.02**	**4.43**	**0.00**	**0.70**	0.0001^†^*
	Log_10_ body mass	0.19	3.31	0.13	10.91	6.00	0.03	0.1285^††^
	Polyandry	0.24	5.15	0.07				
Log_10_ sperm length	1	2.40		<0.001	−21.41	1.00	0.31	0.9999^††^
	**Log_10_ body mass**	−**0.14**	**8.72**	**0.03**	−**22.21**	**0.00**	**0.46**	**0.5456^††^**
	Polyandry	−0.03	3.06	0.13	−19.74	2.00	0.13	0.9999^††^
	Log_10_ Testes mass	−0.05	0.74	0.42	−17.38	5.00	0.04	0.9999^††^
	Log_10_ Testes mass	−0.11	9.41	0.03	−17.80	4.00	0.05	0.6699^††^
	Polyandry	−0.02	2.02	0.21				
	Log_10_ body mass	−0.13	7.49	0.04	−15.19	7.00	0.01	0.9999^††^
	Log_10_ Testes mass	−0.01	0.03	0.87				
Log_10_ spermatophore volume	1	0.53		<0.001	1.34	33.00	0.00	0.0001^††^
	Log_10_ body mass	0.45	4.21	0.09	2.05	34.00	0.00	0.0001^††^
	Polyandry	0.18	7.72	0.03	−0.12	32.00	0.00	0.0001^†^*
	Log_10_ Testes mass	0.70	164.32	<0.001	−20.46	12.00	0.00	0.0001^††^
	Log_10_ sperm length	−2.10	3.61	0.11	2.54	34.50	0.00	0.0001^†^*
	**Log_10_ body mass**	**0.14**	**596.08**	**<0.001**	−**31.96**	**0.00**	**0.99**	**0.0001^††^**
	**Log_10_ Testes mass**	**0.63**	**834.48**	**<0.001**				
	Log_10_ body mass	0.27	6.29	0.05	4.45	36.00	0.00	0.0001^††^
	Polyandry	0.14	3.95	0.10				
	Log_10_ body mass	0.29	3.74	0.45	8.61	41.00	0.00	0.0001^††^
	Log_10_ sperm length	−1.04	0.32	0.59				

The best models for each dependent variable (with higher wt) are shown in bold. Abbreviations: ΔAICc: difference of AICc between the best model and the rest of the models; wt: Akaike weight gives an idea of the chance of the model to be the best model explaining the dependent variable; λ: level of phylogenetic influence on the analyzed variable. The superscripts following λ value indicate significance levels (^†(Cross)^ for P>0.05 (not significant), and *^(asterix)^ for P<0.05) in likelihood ratio tests against models with λ = 0 (first position) and λ = 1 (second position).

#### Sperm length

Sperm length varied from 208±15 µm to 302±3 µm. *Urophonius brachycentrus* together with B. cordubensis had the longest spermatozoa in the dataset (Table 1). On the opposite side, *Br. pentheri* had the shortest sperm, and the rest of the species had similar values oscillating from 233 to 269 µm. There was a significant effect of body mass on sperm length (lsl∼lbm, Table 2). The pendant was negative (b<0) (Fig. 3 B). The allometric equation found was Sperm length = 212.96*body mass^−0.14^.

#### Spermatophore’s volume (Ejaculate volume)

Spermatophore’s trunk volume varied but not as much as testes mass. The greatest difference among analyzed species is of about five times, for example between *T. dorbignyi and Br. pentheri* (see Table 1). The linear logarithmic equation tested was Log_10_ (spermatophore volume)  = 0.72+0.43*Log_10_ (Body mass) (Fig. 3 C). With those parameters, the allometric equation was as follows: Testes mass = 5.22*body mass^0.43^. However, the relationship between testes mass and body mass was marginally not significant (lsv∼lbm, Table 2).

### Statistical Phylogenetic Analysis on Testes Mass and Sperm Package Length in Relation to Levels of Polyandry

#### Models explaining testes mass

Model selection analysis for testes mass, sperm package length and ejaculate volume resulted in one best model for each dependent variable (Table 2, highest wt values). The best-fitting model for testes mass considered only polyandry as predictor with almost 70% of chance of being the best model over 22% of the null model, over 3 times better at explaining ltm (Table 2). The phylogenetically controlled GLS regression analysis showed a positive influence of polyandry on testes mass (Fig. 3D, Table 2). The lambda value for this model was significantly closed to 1 (Table 2).Body mass performed poorly in tested models explaining response variables, as seen the previous allometric analysis (see Fig. 3A).

#### Models explaining sperm length

Model selection analysis on sperm length showed that there is also only one model that best fit this data. In this case, according to the allometric analysis, the model incorporated only body mass and had a 46% chance of being the best model (Table 2). This value was greater than the Akaike’s weight for the null model (1.5 times bigger). There was a negative influence of body mass in sperm package length (Fig. 3 B). Polyandry levels (Fig. 3 E) and Testes mass (both predictors associated with sperm competition) alone were not good predictors of sperm length. Nevertheless, the additive models of these variables with body mass were significant or marginally significant as shown in Table 2. Nevertheless these two models were not selected by model selection analysis.

#### Models explaining spermatophore volume

The best model for explaining ejaculate volume had body mass and testes mass as predictors. This model beared an astonishing chance of being the best model. The Akaike’s weight value was almost 100%. The relationship between spermatophore volume, both body mass and testes mass was highly significant with a positive relationship. Lambda value was not significantly close to 0 or 1. Both relationships with spermatophore volume were positive (See Fig. 3 C and F). Although there were other models with significant values (p<0.05), model selection analysis did not choose them.

## Discussion

In this study we have analyzed the relative importance of body mass and polyandry over testes mass, sperm length and ejaculate volume, as well as some biological important relationships among these last three variables. For accomplishing this task, we used scorpions that represent an ancient sex model. To date, this is the first analysis of this kind performed on arthropods with indirect sperm transfer. The addition of a phylogenetically comparative analysis gives extra support to the findings made. The results are supported by classic sperm competition theories, and some of the patterns are found in other organisms closely related to arachnids as well as distant related groups.

We found that testes mass varied widely among analyzed species, both in relative (GSI) and absolute values. In some cases, the difference was of an order of magnitude across species. This pattern of variation in testes mass has been widely described in similar analyses in other invertebrates (e.g. [21] (butterflies); [61] (fruit flies); [66] (fireflies); [67] (bushcrickets); [9], [68] (Beetles)). In Table S2, we have reviewed some studies that used GSI values for assessing relative testes mass in insects, which represent a well-studied group in this subject. We compared these GSI values with the ones obtained for scorpions. In general, values for scorpions are low, with *Timogenes* species showing the lowest values of the reviewed dataset (less than 1%). *Bothriurus cordubensis* ranks higher in the table but far from the colossal values shown by some bushcrickets (eg. *Sepiana sepium* or *Platycleis affinis*), which are almost 3 times bigger than *B. cordubensis*’ GSI value.

Our results showed that body mass did not influence testes mass. In some insects, a clear positive relationship between body and testes was observed (eg. [21], [61]). Nevertheless, Wedell and Hosken [69] suggest that in fact, there is usually no relationship between both variables (see also [70], [71]), as we found for scorpions in the present study. As predicted, we found a clear positive influence of polyandry (a reliable estimator of sperm competition risk) over testes mass. The positive effect of sperm competition on testes mass have been widely demonstrated in various other taxa (e.g. [21] (butterflies); [25] (anurans); [66] (fireflies); [67] (bushcrickets); [72] (primates); [73] (bats); [74], [75] (birds); [76] (ants); [77] (seed beetle)). The results suggest that testes mass in scorpions would be a reliable predictor of sperm competition risk as suggested by many studies (e.g. [19], [78]). In this scenario, sperm would compete numerically following the fair raffle principle [6], [79], [80]. Increased gonadal investment would be traduced directly to sperm numbers because testes would only produce sperm (following what was suggested for insects, see [81]). The strong positive association between testes mass and spermatophore volume suggests, indeed, that bigger testes produce a bigger volume of ejaculate for transferring to the female. At the moment, we do not know if a greater volume of ejaculate is traduced directly to an increase in sperm number in scorpions. Preliminary analyses in seminal vesicle volume for these species, suggested that sperm volume was positively related to sperm count, (Vrech, unpublished data).

All analyzed scorpions were polyandrous except for *U. brachycentrus*. In this species, females mate only once because males deposit a very efficient mating plug [44], [48]. The presence of this mating plug seems to make females unreceptive and/or unattractive to new males (Romero-Lebrón, Vrech & Peretti, unpublished data). The mating plug in this species should have appeared in the past as a strategy to overcome the risk of sperm competition [45]. Sperm competition should have been huge before the appearance of this strategy. The investment in sperm plug should have favored the reduction of testes mass over evolutionary time. However, from a wide evolutionary perspective, the appearance of an efficient genital plug would be recent in this species [48], (Mattoni and Peretti unpublished data) without a clear optimization in gonadal investment yet.

Similarly, both *Timogenes* species did not show the expected testes mass inferred by their polyandry levels. Unfortunately, we do not know why these species show such small testes mass, but we can speculate there is a phylogenetic component, as both species belong to the same genus.

Some other possible explanations could be given for this unexpected result in *Timogenes species*: a.- Testes could be nonfunctional during some periods of time or they could stop producing sperm after the last molt, as happens in some other arachnids [82], [83]. b.- They could be nearly monandrous in nature, however they behave as polyandrous in controlled laboratory essays. c.- Sex ratio could be biased towards males, although sex ratio in these species are not yet fully evaluated (M. Nime, unplublished data). The study of physiological testes function as well as resource allocation influence over sex ratio (e.g. [84], [85]) in these species could give in the future interesting insights that could delimit better the interpretation of the data of this analysis. For example, the use of different evolutionary strategies on resource allocation for male function either through the increase of testes mass or other traits. However, for this purpose new information from field studies on sex ratio is needed.

### Sperm Transfer, Ejaculate Volume and Sperm Length

The positive effect of body mass on ejaculate volume could be related to the size of the spermatophore itself. Bigger species produce bigger spermatophores with bigger storing capacity. The results suggest that this character scales negatively with body mass, as the pendant *b* is positive but smaller than one [86]. Although, studies show that positive allometry is generally shown in characters under directional sexual selection, one clear exception for this pattern is insect genitalia where negative allometry is usually observed (see [86], reviewed in [87]).

It is important to point out that sperm transfer in scorpions has distinct characteristics. In contrast to what happens in all other arthropods usually analyzed under the sperm competition theory, scorpions transfer their ejaculate using a sclerotized spermatophore deposited on soil [1], [48], [88]. The features implied in this ancient sex model drive to interesting questions at a macroevolutionary level. For example, how sperm competition pressures shaped the use of an indirect mode of sperm transfer. This question is particularly important in scorpions, considering its ancient phylogenetic position. Indeed, it is important to point out that natural selection promoted the appearance of spermatophores as a solution to the desiccation problem of some terrestrial organisms, including scorpions [38], [89], [90]. Nevertheless, sexual selection via sperm competition is directly involved in the evolution of direct sperm transfer, passing first through indirect spermatophore transfer (e.g. scorpions, amblypigids, uropygids) [5], [42], [91], [92]. In resume, spermatophores were the first attempt to put the sperm close to the ova. Spermatophores usually appear in basal systematic position groups, such as scorpions [89], [93], [94]. They are solitary predators that in general show relative low density and require more intimate contact compared to other groups with spermatophores [89], [92]. Spermatophores in scorpions have a relatively fixed volume [52], and this volume varies up to a certain maximum value (Vrech, unpublished data). Therefore, males will face a clear limitation in the ejaculate volume, a fact that would be relevant in a context of sperm competition [1], [48]. Interestingly, this could imply that, even though spermatogenesis is continuous in adulthood [95], [96], the great majority of the sperm production would not be transferred to females during the male’s life. Nevertheless, males could produce several spermatophores during their life [48], [57]. Some preliminary results suggest that scorpion males can adjust the volume of sperm deposited in the spermatophore (D. Vrech, C. I. Mattoni & A. V. Peretti unpublished data), but it is yet unknown if there is a real mechanism of sperm allocation in scorpions.

The volume of the sperm drop was smaller than the volume of the whole spermatophore trunk, as preliminary tested in both *Timogenes* species (Vrech, unpublished data). Volumes of ejaculated sperm and spermatophore’s trunk covary, both showing a positive relationship with testes mass (Vrech unpublished data). This pattern may suggest that bigger testes would be associated with bigger ejaculates. This idea has the support of the sperm competition theory [5], [12] where polyandry promotes bigger testes in males generating more spermatozoa to prevail in a numeric type of competition [6].

The influence of sperm competition risk has been widely tested on spermatozoa length. There is strong support of a positive influence of sperm competition over sperm length (eg. [21] (butterflies); [22] (moths); [23], [33] (fishes); [27], [29], [97] (birds); [32], [98], [99] (mammals); [72] (bats), [100] (Beetle), [101] (fruit flies); [102] (*Coenorabditis elegans*)). However, our results showed no influence of sperm competition risk (measured as the level of female polyandry and testes mass) on total sperm length. This result would agree with those published mostly in vertebrates (e.g. [27], [72], [99], [100]). According to Parker [12], sperm size should not necessary increase with an increase in the risk of sperm competition. Indeed, there must be some selective advantage in increased sperm size. With no advantage, sperm would tend to remain at a minimum size (due to energetic reasons) independent of sperm competition influence [33].

Unlike with testes mass, body mass did influenced sperm length. The negative association found suggests that species with greater body masses had smaller spermatozoa. Similar association is hard to find among invertebrates, but in vertebrates, many studies suggest a positive relationship or no relationship at all (see [21], [22], [30], [101], [102]). Cummins and Woodall [103] found a similar pattern in mammals, but their findings were not supported by posterior analyses [30], [99]. In snakes, Tourmente et al. [26] found a similar negative association between body mass and medial piece length, but found no relationship between total sperm length and body mass. This negative pattern between body mass and sperm size in scorpions could be partially explained by the dilution effect that species with great body volumes usually experiment [104]–[106]. Bearing in mind the hypothetical occurrence of a trade-off between sperm length and number, sperm in scorpions could be small and numerous in bigger species relative to small ones. Females from bigger species show a relative increase in the volume of the genital tract where the ejaculate is distributed [107]. In this situation, males from bigger species should produce a greater amount of smaller spermatozoa to fill the increased volume of the female genital tract [108].

In conclusion, the present study shows a variation in testes mass that was influenced by polyandry but not by body mass. Body mass positively related to ejaculate volume, and negatively to sperm length. Ejaculate volume and testes mass were strongly associated and both increased linearly in the relationship. Some models were under phylogenetic effects but the great majority lacked phylogenetic influence. *Timogenes* species showed disproportionally small testes relative to the expected for their body mass. *Urophonius brachycentrus* produces a very effective mating plug, but testes mass is not as small as would be expected in this situation, suggesting a very strong sperm competition over evolutionary time. Sexual selection mechanisms such as sperm competition are poorly known in scorpions. In the future, studies with a bigger sample of species are strongly needed. Unfortunately, this is no easy task as species should have a well-known mating system, and they should be easy to collect, as fresh material is needed. This last condition is essential as fixed material is difficult and inexactly to use, contrary to what happens for example in vertebrates. Besides, sperm concentration analyses of scorpions are strongly needed to elucidate the real sperm competition tactics affecting these arachnids. Furthermore, a detailed analysis of how sperm competition shapes different components of spermatozoa is also needed. Polyandry levels should be enhanced with more field observations, as well as additional knowledge of population parameters such as operational sex ratio.

## Supporting Information

Table S1 **List of voucher specimens.** All specimens were deposited in the Scientific Collection of the Institute of Animal Diversity and Ecology (IDEA), CONICET- Universidad Nacional de Córdoba, Argentina. In the following list each voucher specimen is described (species name, catalog number, collecting date and site, collectors’ names).
(DOC)

Table S2 **Comparative table reviewing GSI values from different species of invertebrates.** Species are ordered from the smallest to the highest GSI value.
(DOC)
