# Supplementary material for: Testes Mass, but Not Sperm Length, Increases with Higher Levels of Polyandry in an Ancient Sex Model
Source: PLoS One. 2014 Apr 15;9(4):e94135. doi: 10.1371/journal.pone.0094135 (PMC3988103; doi:10.1371/journal.pone.0094135)
Supplement: Table S1 — List of voucher specimens. All specimens were deposited in the Scientific Collection of the Institute of Animal Diversity and Ecology (IDEA), CONICET- Universidad Nacional de Córdoba, Argentina. In the following list each voucher specimen is described (species name, catalog number, collecting date and site, collectors’ names). (DOC) [file pone.0094135.s001.doc]

| ***Species*** | **Catalog number** | **Collecting date** | **Collecting location** | **Collectors** |
| --- | --- | --- | --- | --- |
| *Bothriurus bonariensis* | Male 01 | 20-12-10 | Mendiolaza, Córdoba, Argentina | D Vrech, P Olivero |
| *Bothriurus* *bonariensis* | Male 02 | 20-12-10 | Mendiolaza, Córdoba, Argentina | D Vrech, P Olivero |
| *Bothriurus* *bonariensis* | Male 03 | 20-12-10 | Mendiolaza, Córdoba, Argentina | D Vrech, P Olivero |
| *Bothriurus* *bonariensis* | Male 04 | 22-12-10 | Quintas de Arguello, Córdoba, Argentina | N Pellegrini |
| *Bothriurus* *bonariensis* | Male 05 | 25-12-10 | Reserva Municipal San Martin, Córdoba, Argentina | N Pellegrini |
| *Bothriurus* *bonariensis* | Male 60 | 19-03-11 | Piedras de afilar, Montevideo, Uruguay | D Vrech, P Olivero |
| *Bothriurus* *bonariensis* | Male 66 | 19-03-11 | Piedras de afilar, Montevideo, Uruguay | D Vrech, P Olivero |
| *Bothriurus* *bonariensis* | Male 72 | 20-12-10 | Mendiolaza, Córdoba, Argentina | D Vrech, P Olivero |
| *Bothriurus* *bonariensis* | Male 75 | 20-12-10 | Mendiolaza, Córdoba, Argentina | D Vrech, P Olivero |
| *Bothriurus* *bonariensis* | Male 76 | 20-12-10 | Mendiolaza, Córdoba, Argentina | D Vrech, P Olivero |
| *Bothriurus* *bonariensis* | Male 79 | 20-12-10 | Mendiolaza, Córdoba, Argentina | D Vrech, P Olivero |
|  |  |  |  |  |
| *Bothriurus* *cordubensis* | Male 01 | 30-01-09 | Villa Berna, Córdoba, Argentina | C Mattoni |
| *Bothriurus* *cordubensis* | Male 02 | 30-01-09 | Villa Berna, Córdoba, Argentina | C Mattoni |
| *Bothriurus* *cordubensis* | Male 03 | 30-01-09 | Villa Berna, Córdoba, Argentina | C Mattoni |
| *Bothriurus* *cordubensis* | Male 04 | 30-01-09 | Villa Berna, Córdoba, Argentina | C Mattoni |
| *Bothriurus* *cordubensis* | Male 05 | 30-01-09 | Villa Berna, Córdoba, Argentina | C Mattoni |
| *Bothriurus* *cordubensis* | Male 06 | 30-01-09 | Villa Berna, Córdoba, Argentina | C Mattoni |
|  |  |  |  |  |
| *Bothriurus* *rochensis* | Male 02 | Dec/Ene-2011 | Piedras de Afilar, Montevideo, Uruguay | C Toscano-Gadea |
| *Bothriurus* *rochensis* | Male 03 | Dec/Ene-2011 | Piedras de Afilar, Montevideo, Uruguay | C Toscano-Gadea |
| *Bothriurus* *rochensis* | Male 04 | Dec/Ene-2011 | Piedras de Afilar, Montevideo, Uruguay | C Toscano-Gadea |
| *Bothriurus* *rochensis* | Male 05 | Dec/Ene-2011 | Piedras de Afilar, Montevideo, Uruguay | C Toscano-Gadea |
| *Bothriurus* *rochensis* | Male 06 | Dec/Ene-2011 | Piedras de Afilar, Montevideo, Uruguay | C Toscano-Gadea |
| *Bothriurus* *rochensis* | Male 07 | Dec/Ene-2011 | Piedras de Afilar, Montevideo, Uruguay | C Toscano-Gadea |
| *Bothriurus* *rochensis* | Male 08 | Dec/Ene-2011 | Piedras de Afilar, Montevideo, Uruguay | C Toscano-Gadea |
| *Bothriurus* *rochensis* | Male 09 | Dec/Ene-2011 | Piedras de Afilar, Montevideo, Uruguay | C Toscano-Gadea |
| *Bothriurus* *rochensis* | Male 10 | Dec/Ene-2011 | Piedras de Afilar, Montevideo, Uruguay | C Toscano-Gadea |
| *Bothriurus* *rochensis* | Male 11 | Dec/Ene-2011 | Piedras de Afilar, Montevideo, Uruguay | C Toscano-Gadea |
|  |  |  |  |  |
| *Brachistosternus* *ferrugineus* | Male 05 | 21-12-11 | Parque provincial Chancaní, Córdoba, Argentina | D Vrech, M Nime |
| *Brachistosternus* *ferrugineus* | Male 06 | 21-12-11 | Parque provincial Chancaní, Córdoba, Argentina | D Vrech, M Nime |
| *Brachistosternus* *ferrugineus* | Male 07 | 21-12-11 | Parque provincial Chancaní, Córdoba, Argentina | D Vrech, M Nime |
| *Brachistosternus* *ferrugineus* | Male 08 | 21-12-11 | Parque provincial Chancaní, Córdoba, Argentina | D Vrech, M Nime |
| *Brachistosternus* *ferrugineus* | Male 11 | 21-12-11 | Parque provincial Chancaní, Córdoba, Argentina | D Vrech, M Nime |
| *Brachistosternus* *ferrugineus* | Male A | 19-01-12 | Parque provincial Chancaní, Córdoba, Argentina | D Vrech, M Nime |
| *Brachistosternus* *ferrugineus* | Male B | 19-01-12 | Parque provincial Chancaní, Córdoba, Argentina | D Vrech, M Nime |
| *Brachistosternus* *ferrugineus* | Male C | 19-01-12 | Parque provincial Chancaní, Córdoba, Argentina | D Vrech, M Nime |
| *Brachistosternus* *ferrugineus* | Male D | 19-01-12 | Parque provincial Chancaní, Córdoba, Argentina | D Vrech, M Nime |
| *Brachistosternus* *ferrugineus* | Male E | 19-01-12 | Parque provincial Chancaní, Córdoba, Argentina | D Vrech, M Nime |
| *Brachistosternus* *ferrugineus* | Male F | 19-01-12 | Parque provincial Chancaní, Córdoba, Argentina | D Vrech, M Nime |
| *Brachistosternus* *ferrugineus* | Male G | 18-01-12 | Villa del Soto, Córdoba, Argentina | E Romero-Lebrón |
| *Brachistosternus* *ferrugineus* | Male H | 18-01-12 | Villa del Soto, Córdoba, Argentina | E Romero-Lebrón |
| *Brachistosternus* *ferrugineus* | Male I | 18-01-12 | Villa del Soto, Córdoba, Argentina | E Romero-Lebrón |
| *Brachistosternus* *ferrugineus* | Male J | 18-01-12 | Villa del Soto, Córdoba, Argentina | E Romero-Lebrón |
| *Brachistosternus* *ferrugineus* | Male 20 | 21-12-11 | Parque provincial Chancaní, Córdoba, Argentina | D Vrech, M Nime |
| *Brachistosternus* *ferrugineus* | Male 21 | 21-12-11 | Parque provincial Chancaní, Córdoba, Argentina | D Vrech, M Nime |
| *Brachistosternus* *ferrugineus* | Male 22 | 21-12-11 | Parque provincial Chancaní, Córdoba, Argentina | D Vrech, M Nime |
|  |  |  |  |  |
| *Brachistosternus* *pentheri* | Male 01 | 21-11-08 | Salinas Grandes, Córdoba, Argentina | D Vrech, A Peretti, C Mattoni, M Izquierdo |
| *Brachistosternus* *pentheri* | Male 02 | 21-11-08 | Salinas Grandes, Córdoba, Argentina | D Vrech, A Peretti, C Mattoni, M Izquierdo |
| *Brachistosternus* *pentheri* | Male 03 | 21-11-08 | Salinas Grandes, Córdoba, Argentina | D Vrech, A Peretti, C Mattoni, M Izquierdo |
| *Brachistosternus* *pentheri* | Male 05 | 21-11-08 | Salinas Grandes, Córdoba, Argentina | D Vrech, A Peretti, C Mattoni, M Izquierdo |
| *Brachistosternus* *pentheri* | Male 06 | 21-11-08 | Salinas Grandes, Córdoba, Argentina | D Vrech, A Peretti, C Mattoni, M Izquierdo |
| *Brachistosternus* *pentheri* | Male 07 | 21-11-08 | Salinas Grandes, Córdoba, Argentina | D Vrech, A Peretti, C Mattoni, M Izquierdo |
|  |  |  |  |  |
| *Timogenes dorbignyi* | Male 09 | 21-11-08 | Salinas Grandes Córdoba, Argentina | C Mattoni, P Olivero, A Peretti, D Vrech |
| *Timogenes dorbignyi* | Male 26 | 21-11-08 | Salinas Grandes Córdoba, Argentina | C Mattoni, P Olivero, A Peretti, D Vrech |
| *Timogenes dorbignyi* | Male 28 | 21-11-08 | Salinas Grandes Córdoba, Argentina | C Mattoni, P Olivero, A Peretti, D Vrech |
| *Timogenes dorbignyi* | Male 21 | 21-11-08 | Salinas Grandes Córdoba, Argentina | C Mattoni, P Olivero, A Peretti, D Vrech |
| *Timogenes dorbignyi* | Male 07 | 21-11-08 | Salinas Grandes Córdoba, Argentina | C Mattoni, P Olivero, A Peretti, D Vrech |
| *Timogenes dorbignyi* | Male 03 | 21-11-09 | Reserva provincial Chancaní, Córdoba, Argentina | C Mattoni, D Vrech |
| *Timogenes dorbignyi* | Male 02 | 21-11-09 | Reserva provincial Chancaní, Córdoba, Argentina | C Mattoni, D Vrech |
| *Timogenes dorbignyi* | Male 04 | 06-01-11 | Reserva provincial Chancaní, Córdoba, Argentina | M Nime, D Vrech |
| *Timogenes dorbignyi* | Male 03 | 06-01-11 | Reserva provincial Chancaní, Córdoba, Argentina | M Nime, D Vrech |
|  |  |  |  |  |
| *Timogenes elegans* | Male 07 | 22-12-09 | Salinas Grandes | C Mattoni, A Peretti, D Vrech |
| *Timogenes elegans* | Male 05 | 22-12-09 | Salinas Grandes | C Mattoni, A Peretti, D Vrech |
| *Timogenes elegans* | Male 06 | 22-12-09 | Salinas Grandes | C Mattoni, A Peretti, D Vrech |
| *Timogenes elegans* | Male 17 | 06-01-11 | Reserva provincial Chancaní, Córdoba, Argentina | M Nime, D Vrech |
| *Timogenes elegans* | Male 06 | 06-01-11 | Reserva provincial Chancaní, Córdoba, Argentina | M Nime, D Vrech |
| *Timogenes elegans* | Male 05 | 06-01-11 | Reserva provincial Chancaní, Córdoba, Argentina | M Nime, D Vrech |
| *Timogenes elegans* | Male 16 | 06-01-11 | Reserva provincial Chancaní, Córdoba, Argentina | M Nime, D Vrech |
| *Timogenes elegans* | Male 01 | january-11 | Reserva municipal San Martin, Córdoba, Argentina | N Pelegrini |
| *Timogenes elegans* | Male 35 | 10-02-11 | Reserva provincial Chancaní, Córdoba, Argentina | M Nime, D Vrech |
| *Timogenes elegans* | Male 15 | 06-01-11 | Reserva provincial Chancaní, Córdoba, Argentina | M Nime, D Vrech |
| *Timogenes elegans* | Male 08 | 06-01-11 | Reserva provincial Chancaní, Córdoba, Argentina | M Nime, D Vrech |
| *Timogenes elegans* | Male 14 | 06-01-11 | Reserva provincial Chancaní, Córdoba, Argentina | M Nime, D Vrech |
| *Timogenes elegans* | Male 01 | 06-01-11 | Reserva provincial Chancaní, Córdoba, Argentina | M Nime, D Vrech |
| *Timogenes elegans* | Male 13 | 06-01-11 | Reserva provincial Chancaní, Córdoba, Argentina | M Nime, D Vrech |
| *Timogenes elegans* | Male 10 | 06-01-11 | Reserva provincial Chancaní, Córdoba, Argentina | M Nime, D Vrech |
| *Timogenes elegans* | Male 07 | 06-01-11 | Reserva provincial Chancaní, Córdoba, Argentina | M Nime, D Vrech |
| *Timogenes elegans* | Male 01 | 19-03-11 | Reserva provincial Chancaní, Córdoba, Argentina | M Nime, D Vrech |
| *Timogenes elegans* | Male 02 | 19-03-11 | Reserva provincial Chancaní, Córdoba, Argentina | M Nime, D Vrech |
| *Timogenes elegans* | Male 03 | 19-03-11 | Reserva provincial Chancaní, Córdoba, Argentina | M Nime, D Vrech |
|  |  |  |  |  |
| *Urophonius* *brachycentrus* | Male 01 | 24-06-11 | Tanti, Córdoba, Argentina | M Gonzalez, P Olivero, D Vrech |
| *Urophonius* *brachycentrus* | Male 02 | 24-06-11 | Tanti, Córdoba, Argentina | M Gonzalez, P Olivero, D Vrech |
| *Urophonius* *brachycentrus* | Male 03 | 24-06-11 | Tanti, Córdoba, Argentina | M Gonzalez, P Olivero, D Vrech |
| *Urophonius* *brachycentrus* | Male 01 | 21-05-12 | Tanti, Córdoba, Argentina | M Gonzalez, P Olivero, A Peretti, D Vrech |
| *Urophonius* *brachycentrus* | Male 02 | 21-05-12 | Tanti, Córdoba, Argentina | M Gonzalez, P Olivero, A Peretti, D Vrech |
| *Urophonius* *brachycentrus* | Male 03 | 21-05-12 | Tanti, Córdoba, Argentina | M Gonzalez, P Olivero, A Peretti, D Vrech |
| *Urophonius* *brachycentrus* | Male 04 | 21-05-12 | Tanti, Córdoba, Argentina | M Gonzalez, P Olivero, A Peretti, D Vrech |
| *Urophonius* *brachycentrus* | Male 05 | 21-05-12 | Tanti, Córdoba, Argentina | M Gonzalez, P Olivero, A Peretti, D Vrech |
| *Urophonius* *brachycentrus* | Male 06 | 21-05-12 | Tanti, Córdoba, Argentina | M Gonzalez, P Olivero, A Peretti, D Vrech |
