# Supplementary material for: Testes Mass, but Not Sperm Length, Increases with Higher Levels of Polyandry in an Ancient Sex Model
Source: PLoS One. 2014 Apr 15;9(4):e94135. doi: 10.1371/journal.pone.0094135 (PMC3988103; doi:10.1371/journal.pone.0094135)
Supplement: Table S2 — Comparative table reviewing GSI values from different species of invertebrates. Species are ordered from the smallest to the highest GSI value. (DOC) [file pone.0094135.s002.doc]

| **Species** | **Group** | **GSI** | **Reference** |
| --- | --- | --- | --- |
| ***T. dorbignyi*** | **Invertebrates, scorpions** | **0.0037** | **Present study** |
| ***T. elegans*** | **Invertebrates, scorpions** | **0.0048** | **Present study** |
| *Gampsocleis glabra* | Invertebrate, insects Tettiigonidae | 0.0099 | [67] |
| *Ephippiger ephippiger* | Invertebrate, insects Tettiigonidae | 0.0106 | [67] |
| *Steropleurus stali* | Invertebrate, insects Tettiigonidae | 0.0144 | [67] |
| *Metrioptera roeselii* | Invertebrate, insects Tettiigonidae | 0.0157 | [67] |
| *Onthophagus sloanei* | Invertebrate, insects Coleoptera | 0.0158 | [68][68] |
| *Drosophila micromelanica* | Invertebrate, insects Diptera | 0.0173 | [61] |
| ***U. brachycentrus*** | **Invertebrates, scorpions** | **0.0214** | **Present study** |
| *Onthophagus nuchicornis* | Invertebrate, insects Coleoptera | 0.0218 | [68][68] |
| *Onthophagus auruginosis* | Invertebrate, insects Coleoptera | 0.0224 | [68] |
| ***Br.pentheri*** | **Invertebrates, scorpions** | **0.0230** | **Present study** |
| ***B. rochensis*** | **Invertebrates, scorpions** | **0.0233** | **Present study** |
| ***B. bonariensis*** | **Invertebrates, scorpions** | **0.0238** | **Present study** |
| *Onthophagus nodulifer* | Invertebrate, insects Coleoptera | 0.0247 | [68] |
| *Onthophagus haagi* | Invertebrate, insects Coleoptera | 0.0251 | [68] |
| *Onthophagus alcyonides* | Invertebrate, insects Coleoptera | 0.0253 | [68] |
| *Onthophagus binodis* | Invertebrate, insects Coleoptera | 0.0257 | [60] |
| *Poecilimon veluchianus* | Invertebrate, insects Tettiigonidae | 0.0264 | [67] |
| *Onthophagus gazella* | Invertebrate, insects Coleoptera | 0.0267 | [68] |
| *Onthophagus nigriventris* | Invertebrate, insects Coleoptera | 0.0280 | [68] |
| *Onthophagus cribripennis* | Invertebrate, insects Coleoptera | 0.0286 | [68] |
| *Onthophagus hecate* | Invertebrate, insects Coleoptera | 0.0299 | [68] |
| *Poecilimon affinis* | Invertebrate, insects Tettiigonidae | 0.0300 | [67] |
| *Onthophagus australis* | Invertebrate, insects Coleoptera | 0.0302 | [68] |
| ***Br. ferrugineus*** | **Invertebrates, scorpions** | **0.0307** | **Present study** |
| *Onthophagus vermiculatus* | Invertebrate, insects Coleoptera | 0.0307 | [68] |
| *Onthophagus taurus* | Invertebrate, insects Coleoptera | 0.0322 | [68] |
| *Antaxius pedestris* | Invertebrate, insects Tettiigonidae | 0.0332 | [67] |
| *Tettigonia viridissima* | Invertebrate, insects Tettiigonidae | 0.0337 | [67] |
| *Pholidoptera griseoaptera* | Invertebrate, insects Tettiigonidae | 0.0339 | [67] |
| *Metrioptera brachyptera* | Invertebrate, insects Tettiigonidae | 0.0340 | [67] |
| *Eupholidoptera chabrieri* | Invertebrate, insects Tettiigonidae | 0.0347 | [67] |
| *Onthophagus fuliginosus* | Invertebrate, insects Coleoptera | 0.0356 | [68] |
| *Drosophila melanica* | Invertebrate, insects Diptera | 0.0356 | [61] |
| *Drosophila wassermani* | Invertebrate, insects Diptera | 0.0362 | [61] |
| *Anonconotus alpinus alpinus* | Invertebrate, insects Tettiigonidae | 0.0372 | [67] |
| *Onthophagus rupicapra* | Invertebrate, insects Coleoptera | 0.0397 | [68] |
| *Drosophila nannoptera* | Invertebrate, insects Diptera | 0.0402 | [61] |
| *Decticus verrucivorus* | Invertebrate, insects Tettiigonidae | 0.0438 | [67] |
| *Yersinella raymondi* | Invertebrate, insects Tettiigonidae | 0.0446 | [67] |
| *Drosophila acanthoptera* | Invertebrate, insects Diptera | 0.0453 | [61] |
| *Platycleis albopunctata* | Invertebrate, insects Tettiigonidae | 0.0459 | [67] |
| ***B. cordubensis*** | **Invertebrates, scorpions** | **0.0475** | **Present study** |
| *Drosophila melanogaster* | Invertebrate, insects Diptera | 0.0505 | [61] |
| *Metrioptera saussuriana* | Invertebrate, insects Tettiigonidae | 0.0519 | [67] |
| *Drosophila mojavensis* | Invertebrate, insects Diptera | 0.0584 | [61] |
| *Anonconotus baracunensis* | Invertebrate, insects Tettiigonidae | 0.0649 | [67] |
| *Anonconotus pusillus* | Invertebrate, insects Tettiigonidae | 0.0694 | [67] |
| *Anonconotus ghilianii* | Invertebrate, insects Tettiigonidae | 0.0792 | [67] |
| *Drosophila pachea* | Invertebrate, insects Diptera | 0.0858 | [61] |
| *Drosophila hydei* | Invertebrate, insects Diptera | 0.0938 | [61] |
| *Drosophila eohydei* | Invertebrate, insects Diptera | 0.0941 | [61] |
| *Drosophila bifurca* | Invertebrate, insects Diptera | 0.1060 | [61] |
| *Sepiana sepium* | Invertebrate, insects Tettiigonidae | 0.1353 | [67] |
| *Platycleis affinis* | Invertebrate, insects Tettiigonidae | 0.1376 | [67] |
|  |  |  |  |
|  |  |  |  |
|  |  |  |  |
|  |  |  |  |
|  |  |  |  |
|  |  |  |  |
|  |  |  |  |
|  |  |  |  |
